# More Prevalent and Severe Low Bone-Mineral Density in Boys with Severe Adolescent Idiopathic Scoliosis Than Girls: A Retrospective Study of 798 Surgical Patients

**DOI:** 10.3390/jcm12082991

**Published:** 2023-04-20

**Authors:** Zhichong Wu, Xiufen Zhu, Leilei Xu, Zhen Liu, Zhenhua Feng, Vivian Wing Yin Hung, Jack Chun Yiu Cheng, Yong Qiu, Wayne Y. W. Lee, Tsz Ping Lam, Zezhang Zhu

**Affiliations:** 1 Division of Spine Surgery, Department of Orthopedic Surgery, Nanjing Drum Tower Hospital, The Affiliated Hospital of Nanjing University Medical School, Nanjing 210008, China; 2 Joint Scoliosis Research Center of The Chinese University of Hong Kong and Nanjing University, Hong Kong, China; 3 Osteoporosis and Metabolic Bone Disease Center, Department of Orthopedic Surgery, Nanjing Drum Tower Hospital, The Affiliated Hospital of Nanjing University Medical School, Nanjing 210008, China; 4 Department of Orthopaedics and Traumatology, The Chinese University of Hong Kong, Hong Kong Special Administrative Region, Hong Kong, China; 5 SH Ho Scoliosis Research Laboratory, Faculty of Medicine, The Chinese University of Hong Kong, Hong Kong Special Administrative Region, Hong Kong, China

**Keywords:** adolescent idiopathic scoliosis, sexual dimorphism, bone mineral density, risk factors

## Abstract

Introduction: A total of 0.1–0.8% of AIS patients progress to severe stages without clear mechanisms, and AIS girls are more prone to curve progression than boys. Recent studies suggest that AIS girls have systemic and persistent low bone-mineral density (BMD), which has been shown to be a significant prognostic factor of curve progression in AIS. The present study aimed to (a) investigate the prevalence of low BMD in patients with severe AIS and (b) assess the sexual dimorphism and independent risk factors of low BMD in severe AIS patients. Materials and Methods: A total of 798 patients (140 boys vs. 658 girls) with AIS who reached surgical threshold (Cobb ≥ 40°) were recruited. BMD were assessed using BMD Z-scores from dual-energy X-ray absorptiometry (DXA). Demographic, clinical, and laboratory values of the subjects were collected from their medical records. Logistic regression analysis was performed to identify independent risk factors of low BMD. Results: The overall prevalence of BMD Z-score ≤ −2 and ≤ −1 were 8.1% and 37.5%, respectively. AIS boys had significantly lower BMD Z-scores (−1.2 ± 0.96 vs. −0.57 ± 0.92) and higher prevalence of low BMD (Z-score ≤ −2: 22.1% vs. 5.2%, *p* < 0.001; Z-score ≤ −1: 59.3% vs. 32.8%, *p* < 0.001) than girls. Sex, BMI, serum alkaline phosphatase, and potassium were independent factors of low BMD in the severe AIS patients. Conclusions: The present large cohort of surgical AIS patients revealed that low BMD is more prevalent and severe in boys than in girls with severe curves. Low BMD may serve as a more valuable predictive factor for curve progression to the surgical threshold in boys than girls with AIS.

## 1. Introduction

Adolescent idiopathic scoliosis (AIS) is a complex three-dimensional deformity of the spine with unknown causes [1]. Being the most common type of scoliosis, AIS affects 2–4% of primary school children [2,3]. A total of 0.1–0.8% of AIS patients progressed to severe stages and may require invasive surgery. Girls with AIS are shown to be more vulnerable to curve progression than boys without clear pathological mechanisms. Recent studies have suggested that 24–38% AIS girls have a systemic low bone-mineral density (BMD), which has been demonstrated to be a significant prognostic factor of curve progression. Moreover, longitudinal studies revealed that low-normal BMD status in AIS girls can persist beyond skeletal maturity. However, the prevalence of low BMD in AIS boys and in severe AIS patients remains largely undefined.

Puberty is a critical period of bone formation and skeletal development. Up to 50–60% of peak bone mass is accrued during puberty, primarily within 2 years around growth spurts [4,5]. Failure to reach optimal peak bone mass at the end of growth will result in an increased future risk of osteoporosis and fractures [6]. Ohashi et al. reported that 11 of 21 middle-aged AIS patients who underwent spinal surgery with more than 20-year follow-up had osteopenia or osteoporosis [7]. Considering the high prevalence and clinical impact of decreased BMD in AIS patients, it is necessary for clinicians to assess bone health in adolescents at risk of low BMD to optimize bone mass accumulation, predict curve progression, and engage in future osteoporosis prevention.

Dual-energy X-ray absorptiometry (DXA) is not readily available and that there are currently no evidence-based guidelines for the appropriate screening, monitoring, and treatment of low BMD in AIS patients, especially for AIS boys and for those with severe curves. Therefore, we conducted this retrospective study for AIS patients with severe curves, aiming to (a) investigate the prevalence of low BMD in patients with severe AIS, (b) assess the sexual dimorphism of BMD in severe AIS, and (c) identify independent risk factors of low BMD in severe AIS. Furthermore, the most severe AIS patients will require spinal surgery, which may lead to significant blood loss, especially during the procedure of transpedicular screw placement. Whether BMD status influences blood loss during surgery remains unknown; therefore, the association of BMD with intraoperative blood loss was also investigated in this study.

## 2. Methods

### 2.1. Study Population

Patients were recruited if they were diagnosed with AIS, had Cobb angles greater than 40 degrees, and had received DXA scans before surgery in our center. Patients were excluded if they were not of Chinese Han origin or had a previous surgery history. Two experienced senior surgeons (Y. Qiu and Z. Zhu) made the diagnosis for the participants. A total of 798 AIS patients, aged between 10–18 years, were recruited consecutively from May 2016 to October 2021. This retrospective study was approved by the Affiliated Drum Tower Hospital of Nanjing University Medical School (2021-LCYJ-DBZ-05). The study design was in accordance with relevant rules and regulations. 

### 2.2. BMD Evaluation

BMD (g/cm^2^) was evaluated in all subjects using GE Lunar DXA scanners (Prodigy or iDXA; GE Healthcare, Waukesha, WI, USA) according to the standard procedure. The lumbar spine (L1-L4) and hips (femoral neck and total hip) were selected as regions of interest (ROI), as recommended by the International Society for Clinical Densitometry (ISCD) [8]. Given that the spine deformity in the patients may lead to overestimation of BMD values [9], the left femoral neck was used to define bone health, as previously reported [10]. The BMD Z-scores of the patients, defined by standard deviations away from the mean BMD values, were generated according to the normative values of age- and sex-matched healthy East Asian adolescents [11,12]. 

### 2.3. Assessment of Potential Risk Factors

BMD data, anthropometric measurements, and laboratory values, which were evaluated in our scoliosis center, were obtained from participants’ medical records. Clinical variables under investigation included age, sex, body mass index (BMI), curve type, and Cobb angle. Items of laboratory examination, which were routinely evaluated before surgery, were also considered as potential predictors of low BMD. The BMI Z-score was calculated based on the WHO age- and sex-specific growth references for adolescents [13].

### 2.4. Evaluation of Intraoperative Blood Loss

Patients who underwent posterior spinal surgery were included in the evaluation of intraoperative blood loss. Exclusive criteria included history of SRS-Schwab III-VI grades of osteotomy, which is usually associated with a higher degree of blood loss [14]. All patients were anesthetized and operated by the same team. Total blood loss (TBL) was calculated by the total amount of intraoperative suction minus the volume of total fluid used for irrigation. Normalized blood loss (NBL) was calculated as (total blood loss)/(weight * numbers of fused levels) [15].

### 2.5. Statistical Analysis

Data are presented as means ± standard deviation (SD) or counts (percentages). For the inter-group comparison, two-tailed Student’s *t*-test was used for continuous variables, except for Cobb angle, which was compared by Mann–Whitney U test due to skewed distribution. Chi-squared tests were performed for categorical variables, such as sex, curve pattern, and prevalence of low BMD. Multiple linear regression analysis was used to determine the relationship between clinical characteristics and BMD Z-scores; girl and boy sex were set as 0 and 1 in the analysis, respectively. Logistic regression was used to identify potential independent risk factors of low BMD. Area under the curve (AUC) of receiver operating characteristic (ROC) was used to compare the discriminative ability of risk factors for low BMD. Pearson’s correlation analysis was performed to investigate the correlation between BMD and intraoperative blood. All statistical analyses were conducted with SPSS (v.19.0) software, and all data were graphed using Graphpad Prism v8.3. *p* < 0.05 was considered statistically significant.

## 3. Results

### 3.1. Clinical Characteristics

**Figure 1:**
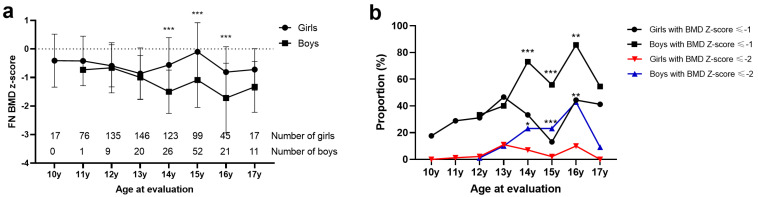
The mean value and standard error of femoral neck bone mineral density (BMD) (**a**) and proportion of low BMD (**b**) in AIS boys and girls. Subjects aged between 10–11 y were divided into the 10 y group,; the same criterion was used for other age groups; * indicates *p* < 0.05, ** indicates *p* < 0.01, *** indicates *p* < 0.001. Student’s *t*-test was used for (**a**), and the chi-squared test was used for (**b**).

Table 1 presents the baseline characteristics of the cohort. Of 798 patients, 140 (17.5%) were boys. The mean age of the subjects was 14.0 years, the mean height was 161.9 cm (Z-score 0.41), the mean BMI was 18.2 kg/m^2^ (Z-score −0.65), and the mean Cobb angle was 53.8 degrees. Boys were older than girls, and they had different curve patterns. However, there was no significant difference in terms of BMI Z-score or Cobb angle between boys and girls. The mean femoral neck BMD Z-score was −0.68, 37.5% of overall AIS patients had a BMD Z-score of ≤−1, and 8.1% of patients had a BMD Z-score of ≤−2. Boys had a higher proportion of BMD Z-scores of ≤−1 (59.3% vs. 32.8%, OR = 5.2, *p* < 0.001) and BMD Z-scores of ≤−2 (22.1% vs. 5.2%, OR = 3.0, *p* < 0.001) compared with girl patients. Subgroup analysis showed that BMD Z-score in AIS boys was lower than AIS girls in each age group, and AIS boys had a significantly higher prevalence of low BMD at 14, 15, and 16 years of age than AIS girls (Figure 1). After adjustment for age, Cobb angle, and BMI Z-score by multiple linear regression analysis, sex remained significant (Beta = −0.288, *p* < 0.001), supporting its remarkable effect on the bone mineral density of AIS patients.

### 3.2. Risk Factors of Low BMD in AIS Patients

Considering the clinical importance of low BMD, we focused on investigating the independent risk factors of low BMD (BMD Z-score ≤ −2 and BMD Z-score ≤ −1) in severe AIS patients. Patients with and without low BMD (Z-score ≤ −2 and −1) were compared in terms of baseline characteristics, disease information, and laboratory values (Table 2). Patients with BMD Z-scores of ≤−2 were significantly older (14.7 vs. 13.9 years, *p* < 0.001), had a higher proportion of boys (47.7% vs. 14.9%, *p* < 0.001), and had a lower BMI Z-score (−1.6 vs. −0.16, *p* < 0.001) than patients with BMD Z-scores of >−2. They also had an increased concentration of alkaline phosphatase and total bilirubin. A similar trend was observed in patients with or without BMD Z-scores of ≤−1 (Table 2). Besides the above parameters, patients with BMD Z-scores of ≤−1 also had a larger Cobb angle, a higher concentration of calcium, and a lower concentration of potassium.

**Figure 2:**
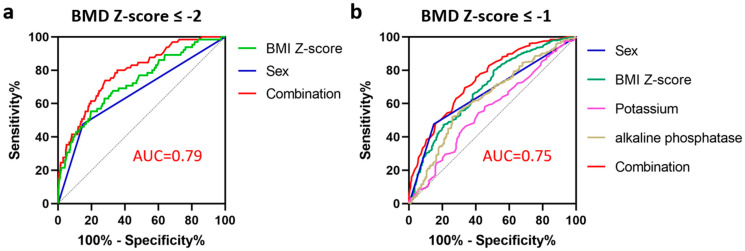
Receiver operating characteristic (ROC) curves of individual parameters or combination of the parameters when predicting BMD Z-scores of ≤−2 (**a**) and BMD Z-scores of ≤−1 (**b**); Area under the ROC curve (ROC) was 0.79 and 0.75 for BMD Z-scores of ≤−2 and Z-scores of ≤−1, respectively.

A logistic regression analysis was then performed to ascertain the independent effects of sex, age, BMI Z-score, and significant laboratory values on low BMD. Boy sex (OR = 5.27, *p* < 0.001) and BMI Z-score (OR = 0.47, *p* < 0.001), but no biochemical parameters, were revealed to be independent predictors of BMD Z-score of ≤−2 (Table 3). Based on the ROC curve analysis, the risk discrimination by the combination of sex and BMI Z-score was good (AUC, 0.79; 95% CI, 0.73–0.84; *p* < 0.001) (Figure 2a). As for BMD Z-scores of ≤−1, sex (OR of boy = 2.39, *p* = 0.005), BMI Z-score (OR = 0.54, *p* < 0.001), potassium (OR = 0.39, *p* = 0.01), and alkaline phosphatase (OR = 1.01, *p* < 0.001) were shown to be significant (Table 4). A combination of the significant parameters also revealed good discrimination ability for BMD Z-scores of ≤−1 (AUC, 0.75; 95% CI, 0.70–0.79; *p* < 0.001) (Figure 2b).

### 3.3. Association of Low BMD with Intraoperative Blood Loss

Most severe AIS patients will require spinal surgery, which may lead to massive blood loss, especially during the procedure of transpedicular screw placement. The association between BMD and intraoperative blood loss remains undefined. To further investigate the potential effect of bone mineral density on blood loss during scoliosis correction surgery, we also examined the correlation between BMD and intraoperative blood loss (IBL). A total of 774 patients were included in the analysis. The average IBL was 874.2 mL, the mean number of levels fused was 9.3, and the mean normalized blood loss (NBL) was 2.1 mL/kg. Patients with low BMD (BMD Z-score ≤ −2) were found to have significantly higher total IBL (1094 ± 818 vs. 855 ± 487 mL, *p* = 0.028) and NBL (2.6 ± 1.4 vs. 2.0 ± 1.1 mL, *p* = 0.001). A significant inverse correlation was observed between femoral neck BMD Z-score and TBL (r = −0.10, *p* = 0.008) or NBL (r = −0.17, *p* < 0.001). Lumbar spine BMD Z-score was stronger correlated with TBL and NBL than femoral neck BMD (r = −0.12, *p* = 0.001 and r = −0.20, *p* < 0.001, respectively).

## 4. Discussion

Although multiple studies have been performed to evaluate bone status in AIS patients, only girls were included in nearly all the previous studies [10,16,17,18]. For the first time, to the best of our knowledge, our data showed sex differences in terms of BMD in AIS patients, which can serve as a significant and independent predictor of low BMD. AIS boys had a significantly lower BMD Z-score and were more likely to have BMD Z-scores of ≤−2 (OR = 5.2) or Z-scores of ≤−1 (OR = 3.0). Sex differences in BMD are an interesting phenomenon that have been reported in patients with several different disorders. Lo et al. [19] reported that male sex was a protective factor for osteopenia and osteoporosis in patients with inflammatory bowel disease. However, in cystic fibrosis, male sex was associated with low BMD Z-scores [20,21]. In the current study, AIS boys had significantly lower standardized Z-scores than girls, which means that BMD in AIS boys deviated more from the mean value of BMD in sex- and age-matched healthy controls than in AIS girls. The reason why low BMD was more common in AIS boys remains unknown. Given that low BMD has been proposed as the primary etiology of adolescent idiopathic scoliosis [17], one potential explanation is that boys have greater spinal stability than girls, as indicated by less posteriorly inclined spines [22], larger vertebral body sizes [23] and less spinal motion [24], thus requiring lower BMD Z-scores to reach the threshold for spinal instability and development of scoliosis. Another possible explanation is that boys are less prone to curve progression [25]; the boys with severe curves may have higher degrees of bone mass derangement, hence lower BMD. In brief, our findings provide evidence that AIS boys might require specific attention due to their lower BMD in puberty, which may serve as a valuable predictor of prognosis that has not been taken seriously before.

Our study also revealed that low BMI Z-score was independently associated with a low BMD Z-score in AIS patients; these results were consistent with previous reports in the AIS population. BMI has been proposed to be the key contributor to BMD from healthy adolescents to postmenopausal women [26]. The potential mechanism underlying the relationship between BMI and BMD may be that higher BMI can produce more gravity and contraction stress on the bones, while the physical stimulation can promote osteogenesis and the development of BMD and bone strength. These findings not only unveil the predictors of low BMD but also highlight the importance of weight gain through good nutrition and adequate physical activity on the improvement of BMD in AIS patients. Besides BMI, serum levels of alkaline phosphatase and potassium were also revealed to be predictors of low BMD. Alkaline phosphatase has been reported to be a marker of osteoporosis among adults, especially postmenopausal women [27,28]. Herein, we add to the literature that it may also be associated with BMD in adolescents. The effect of potassium on BMD has been investigated over the past decades, but the results are inconsistent. Jehle et al. [29] showed that treatment with potassium citrate for 2 years significantly increased BMD in elderly healthy participants without osteoporosis, while another similar study [30] among postmenopausal women failed to show such an association. A recent nationwide study [31] revealed that dietary intake of potassium was positively associated with BMD in both men aged more than 50 years and postmenopausal women. In the present study, we observed that serum level of potassium was an independent preventive factor (OR = 0.37) of low BMD (Z-score ≤ −1), which suggests that higher intakes of potassium may be beneficial to the bone health of AIS patients. 

In previous studies, the prevalence of BMD Z-scores of ≤−1 was reported to range from 29.6% to 33.9%, and the prevalence of BMD Z-scores of ≤−2 ranged from 5.8% to 9.9% [10,17]. The higher proportion of low BMD in our study may be due to the inclusion of boy patients and only severe AIS patients. Recognition of low BMD and optimization of bone mass accrual in severe AIS patients is extremely important, because most patients with severe curves require surgeries to correct spinal deformity, while lower BMD is associated with higher intraoperative blood loss. Furthermore, the long lever-arm of the fused spine can increase stress on the adjacent segments, which may increase the risks of new vertebral fractures. In adult spine deformity, osteoporosis has been reported to be associated with more surgical complications, including proximal junctional failure and screw loosening [32]. Given that osteopenia in AIS can persist into adulthood, low BMD may predispose patients to a higher likelihood of future mechanical complications. Despite this rationale, there is a lack of established guidelines for managing scoliosis-related low BMD. The general principles of physical activity and nutritional supplements for ameliorating bone health can be followed. Physical activity refers to weight-bearing and resistance exercises, and supplements of vitamin D and calcium have been proven to be effective in improving the BMD of AIS girls [33,34]. Medications may also be considered in a few patients with extremely low BMD. Although our results were supportive of higher intakes of potassium for the AIS patients, further studies are warranted to confirm our observations. 

Severe AIS patients typically require spinal surgery, which can result in significant blood loss. The blood loss may cause perioperative cardiovascular and metabolic issues, as well as an increased risk of non-neurological postoperative complications such as respiratory problems, wound infections, and hematomas [35]. Previous studies [35,36] have identified various risk factors associated with intraoperative blood loss, including Risser sign, Cobb angle, BMI, biochemical parameters, and fusion levels. During procedures like transpedicular screw placement and scoliosis correction, low bone-mineral density (BMD) may increase blood loss. Despite this, no research has directly investigated the relationship between BMD and intraoperative blood loss. This study is the first to find that preoperative BMD is inversely related to intraoperative blood loss, with lumbar spine BMD being more strongly correlated with total and normalized blood loss than femoral neck BMD. This finding is reasonable given that all surgical procedures were performed in the spine region. Furthermore, patients with low BMD may experience a 1.3-fold increase in total and normalized blood loss compared to those without low BMD, underscoring the importance of improving preoperative BMD to decrease intraoperative blood loss.

The present study was characterized by a relatively large sample size and the inclusion of boy patients. Several limitations of the study need to be addressed. First, only AIS patients with severe curves were included, so generalizing our conclusions to the whole AIS population requires caution. Second, indications for DXA examination were not documented in their medical record, which may lead to selection bias. Lack of healthy controls is another limitation of our study, and bone mineral density in healthy children and adolescents can vary due to factors such as age, sex, and race [37]. To address this, we utilized Asian-, age-, and sex-specific reference curves for BMD to calculate BMD Z-scores. Still, the incorporation of additional healthy controls may help in better interpretation of the prevalence of low BMD in AIS patients. Lastly, since we have no information on the vitamin D status, physical activity, and dietary information of the subjects, the effects of the factors on BMD remained undefined.

## 5. Conclusions

In conclusion, the overall prevalence of BMD Z-scores of ≤−1 and BMD Z-scores of ≤−2 in severe AIS patients were 37.5% and 8.1%, respectively. AIS boys had a significantly higher proportion of low BMD when compared with girls. Boy sex, lower BMI Z-score, serum alkaline phosphatase, and potassium were independent predictors for low BMD in the subjects. Screening and early intervention for low BMD can be considered as prophylactic measures to mitigate intraoperative blood loss and future mechanical complications.

## Figures and Tables

**Table 1:** Demographic data and information of bone mineral density in girl and boy AIS patients.

	Total (*n* = 798) Mean ± SD *	Girls (*n* = 658)	Boys (*n* = 140)	*p*-Value *
Age, y	14.0 ± 1.6	13.8 ± 1.6	15.1 ± 1.3	<0.001
Height, cm	161.9 ± 8.5	160.0 ± 7.0	171.0 ± 9.1	<0.001
Height Z-score	0.41 ± 1.0	0.41 ± 1.0	0.41 ± 1.1	0.99
BMI, kg/m^2^	18.2 ± 1.9	18.1 ± 2.7	18.9 ± 3.5	0.01
BMI Z-score	−0.65 ± 1.2	−0.64 ± 1.1	−0.69 ± 1.5	0.66
Cobb angle, degrees	53.8 ± 11.3	53.7 ± 11.3	54.0 ± 11.4	0.66
Curve pattern				0.017
Lenke 1, *n*	435	368	67	
Lenke 2, *n*	75	51	24	
Lenke 3, *n*	37	29	8	
Lenke 4, *n*	6	4	2	
Lenke 5, *n*	208	174	34	
Lenke 6, *n*	37	32	5	
Femoral neck BMD, g/cm^2^	0.82 ± 0.12	0.81 ± 0.12	0.85 ± 0.12	0.001
Femoral neck BMD Z-score	−0.68 ± 0.96	−0.57 ± 0.92	−1.2 ± 0.96	<0.001
BMD Z-score ≤ −2, %	8.1	5.2	22.1	<0.001
BMD Z-score ≤ −1, %	37.5	32.8	59.3	<0.001

*p*-value * indicates *p*-value of the comparison between AIS girls and boys. Mean ± SD * indicates that continuous variables were presented as mean ± standard deviation (SD). BMI, body mass index; BMD, bone mineral density. Age, height, BMI, BMD were compared by Student’s *t*-test; Cobb angle was compared by Mann–Whitney U test. Curve pattern and the prevalence of low BMD were compared by chi-squared test.

**Table 2:** Univariate assessment for low BMD (Z-score ≤ −2 and Z-score ≤ −1) in patients with scoliosis.

	Z-Score ≤ −2 (*n* = 65)	Z-Score > −2 (*n* = 733)	*p*-Value *	Z-Score ≤ −1 (*n* = 299)	Z-Score > −1 (*n* = 499)	*p*-Value ^#^
Baseline characteristics						
Age, y	14.7 ± 1.3	13.9 ± 1.6	0.001	14.2 ± 1.6	13.9 ± 1.6	0.015
Boy, *n*	31 (22.1%)	109 (77.9%)	<0.001	83 (59.3%)	57 (40.7%)	<0.001
Girls, *n*	34 (5.2%)	624 (94.8%)		216 (32.8%)	442 (67.2%)	
BMI, kg/m^2^	16.7 ± 2.3	18.4 ± 2.9	<0.001	17.3 ± 2.5	18.8 ± 2.9	<0.001
BMI Z-score	−1.6 ± 1.3	−0.6 ± 1.1	<0.001	−1.1 ± 1.2	−0.36 ± 1.1	<0.001
Cobb angle	54.2 ± 12.8	53.7 ± 11.2	0.99	54.9 ± 12.2	53.1 ± 10.7	0.04
Femoral neck BMD, g/cm^2^	0.66 ± 0.06	0.83 ± 0.11	<0.001	0.72 ± 0.07	0.88 ± 0.10	<0.001
Femoral neck BMD Z-score	−2.4 ± 0.4	−0.5 ± 0.8	<0.001	−1.6 ± 0.5	−0.1 ± 0.7	<0.001
Laboratory values						
Total calcium, mmol/L	2.47 ± 0.14	2.43 ± 0.12	0.051	2.44 ± 0.13	2.43 ± 0.12	0.32
Phosphorous, mmol/L	1.46 ± 0.14	1.47 ± 0.16	0.50	1.49 ± 0.16	1.47 ± 0.16	0.020
Potassium, mmol/L	4.05 ± 0.28	4.12 ± 0.28	0.11	4.08 ± 0.27	4.13 ± 0.28	0.023
Alkaline phosphatase, U/L	172.8 ± 58.6	148.3 ± 72.9	0.027	170.5 ± 77.5	137.5 ± 65.2	<0.001
Albumin, g/L	43.1 ± 2.8	42.4 ± 2.6	0.10	42.6 ± 2.9	42.4 ± 2.3	0.33
Total cholesterol, mmol/L	3. 51 ± 0.71	3.65 ± 0.67	0.16	3.60 ± 0.64	3.67 ± 0.69	0.27
Triglyceride, mmol/L	0.75 ± 0.21	0.82 ± 0.38	0.05	0.81 ± 0.30	0.81 ± 0.40	0.95
Total bilirubin, umol/L	11.91 ± 6.34	9.29 ± 4.56	0.009	10.2 ± 5.1	9.1 ± 4.6	0.007
Creatinine, umol/L	51.5 ± 13.4	47.5 ± 9.6	0.056	47.8 ± 11.0	47.9 ± 9.3	0.92
Total bile acid, umol/L	4.65 ± 3.61	4.70 ± 3.69	0.93	4.9 ± 4.1	4.6 ± 3.4	0.31

*p*-value * indicates the *p*-value of the comparison between the groups with and without BMD Z-scores of ≤−2. *p*-value ^#^ indicates the *p*-value of the comparison between the groups with and without BMD Z-scores of ≤−1. The continuous variables were presented as mean ± standard deviation (SD), and categorical variables were presented as counts (percentages). BMI, body mass index; BMD, bone mineral density. Age, BMI, BMD, and laboratory values were compared by Student’s *t*-test; Cobb angle was compared by Mann–Whitney U test. Sex was compared by chi-squared test.

**Table 3:** Logistic regression analysis for independent predictors of BMD Z-score ≤ −2.

Variables	OR (95% CI)	*p*-Value
Age	0.97 (0.71–1.33)	0.86
Gender		
Girl	1	
Boy	5.27 (2.17–12.8)	<0.0001
BMI Z-score	0.47 (0.34–0.65)	<0.0001
Laboratory values		
Alkaline phosphatase, U/L	1.00 (0.996–1.007)	0.66
Total bilirubin, umol/L	1.03 (0.97–1.10)	0.36

**Table 4:** Logistic regression analysis for independent predictors of BMD Z-scores of ≤−1.

Variables	OR (95% CI)	*p*-Value
Age	1.14 (0.95–1.37)	0.16
Cobb	1.02 (0.998–1.03)	0.09
Gender		
Girl	1	
Boy	2.43 (1.32–4.48)	0.005
BMI Z-score	0.54 (0.45–0.66)	<0.001
Laboratory values		
Alkaline phosphatase, U/L	1.008 (1.004–1.012)	<0.001
Total bilirubin, umol/L	0.99 (0.95–1.04)	0.68
Potassium, mmol/L	0.37 (0.17–0.79)	0.01
Phosphorus, mmol/L	0.75 (0.18–3.13)	0.70

## Data Availability

The datasets used in the present study are not publicly available but are available from the corresponding author upon reasonable request.
